# Effect of blood meal digestion and DNA extraction protocol on the success of blood meal source determination in the malaria vector *Anopheles atroparvus*

**DOI:** 10.1186/1475-2875-12-109

**Published:** 2013-03-21

**Authors:** Josué Martínez-de la Puente, Santiago Ruiz, Ramón Soriguer, Jordi Figuerola

**Affiliations:** 1 Estación Biológica de Doñana (EBD-CSIC). C/Américo Vespucio, Seville, s/n, E-41092, Spain; 2 Diputación de Huelva, Área de Medio Ambiente, Huelva, Spain

**Keywords:** *Anopheles atroparvus*, COI, *Culex*, Malaria, Mosquitoes, PCR, Transmission network

## Abstract

**Background:**

Host identification is an essential step in studies on the transmission dynamics of vector-borne disease. Nowadays, molecular tools allow the identification of vertebrate hosts to the species level. However, the proportion of successful identifications is variable and may be affected by the quality of the samples and the laboratory protocols. Here, the effect of two of these factors, namely the digestion status of mosquito blood meal and the DNA extraction procedure, on the success of host identification by amplification and sequencing of a fragment of the cytochrome oxidase 1 gene were tested.

**Methods:**

Mosquitoes collected both outdoors and indoors during 2012 in southern Spain were identified to species level and their blood meal digestion status recorded using the Sella score, a visual estimation of the digestion status of mosquito blood meals. Each mosquito was assigned randomly to one of two DNA extraction procedures: the quick and cheap HotSHOT procedure or the QIAGEN DNeasy Blood and Tissue® kit and their hosts identified by a molecular method.

**Results:**

Three hundred and forty-seven blood-fed mosquitoes belonging to *Anopheles atroparvus* (n=171), *Culex perexiguus* (n=84), *Culex pipiens* (n=43), *Culex theileri* (n=39), *Culex modestus* (n=5), *Ochlerotatus caspius* (n=4), *Culiseta* sp. (n=1) were included in this study. Overall, hosts were identified from 234 blood meals compromising at least 25 species including mammals, birds and a single reptile. The success of host identification was lower in mosquitoes with an advanced stage of blood meal digestion and for blood meals extracted using the HotSHOT procedure.

**Conclusions:**

The success of host identification decreases with the advanced stage of mosquito blood meal digestion, from 84.5% for recent blood meals to 25.0% for more digested ones. Using the QIAGEN kit, the identification success improved by 17.6%, with larger increases at more advanced stages of blood meal digestion. Availability of blood-fed females used to be very limited for studies of vector ecology, and these results may help to increase the efficiency of blood meal analyses. In addition, results obtained in this study clearly support that the potential malaria vector *An*. *atroparvus* feeds on animals located outdoors but use human-made shelters for resting after feeding.

## Background

The identification of vertebrate feeding sources and host preferences is essential for studies of the dynamics of transmission of vector-borne pathogens. Traditional serological techniques, including precipitin tests and enzyme-linked immunosorbent assays (ELISA), have been used to identify hosts from a diversity of insect vectors [1-3]. Although the use of these methods has provided valuable information, they have several limitations, including the difficulties of obtaining specific antisera against a broad diversity of host species and thereby failing to detect any host being investigated. To solve these limitations, researchers have progressively incorporated molecular approaches based on the amplification of DNA to identify hosts to species level [4].

Successful identification of hosts by PCR-based methods may be limited by the quality and quantity of the host´s DNA contained in the abdomen of mosquitoes [5]. After feeding, the digestion of blood meal in the insect gut favours a quick degradation of host DNA. Therefore, as the stage of blood digestion increases the success of identification of blood meal sources may decrease [6]. Although variable, according to the method employed and the insect species tested, studies in the laboratory have shown that amplification of host DNA fails a few days after feeding [7]. An advance digestion status of blood-fed mosquitoes may be a potential reason explaining the proportion of unidentified blood meals in molecular studies [8-10]. To reduce the potential failure of host identification and the cost derived from these analyses, some studies on field-caught insects, from which the period between feeding and capture is unknown, only include fully engorged females [11,12] or females containing a recent blood meal [13]. However, capturing blood-fed females is a difficult task because they are not attracted to CO_2_ or other commonly used attractants for mosquitoes [14]. Although other more specific techniques for blood-fed female capture, such as resting boxes or aspirations at resting areas may be used, the number of blood-fed females available for analysis is usually limited. For example, in a recent study on mosquitoes, of the total of 212,987 specimens captured, only 911 (0.43%) engorged females produced a successful amplification [9]. Similar results have been also reported on studies on other haematophagous insects, such as *Culicoides*[15,16]. Consequently, it is important to describe protocols for blood meal analysis that maximize amplification success.

Here, the impact of blood meal digestion status and two commonly used DNA extraction protocols on mosquito blood meal identification using DNA sequencing were analysed. Mosquito species studied here have sanitary and ecological importance as potential vectors of pathogens to humans, livestock and wildlife. This is the case for *Anopheles atroparvus*, the primary vector of human malaria in Spain in the past, which has recently been incriminated in a case of autochthonous malaria transmission [17], and different *Culex* species involved in the transmission of avian malaria [18,19] and West Nile and Usutu virus [20].

## Methods

### Study area

**Figure 1:**
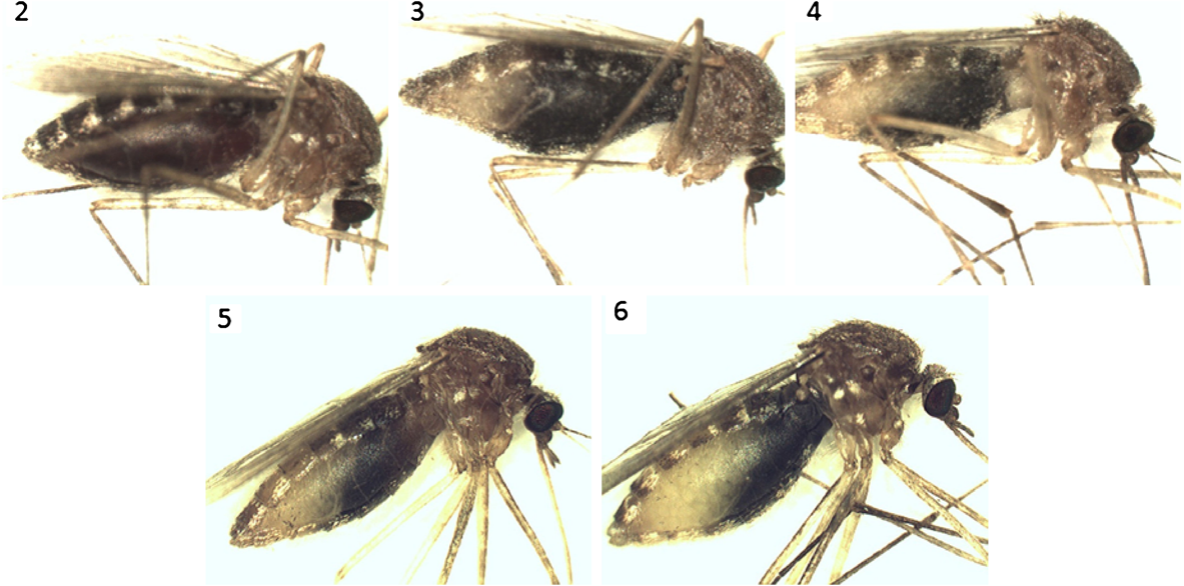
***Culex pipiens *females with different stages of blood meal digestion.** Numbers indicate the stage of blood meal digestion according to the Sella´s score.

Mosquito captures were done at Cañada de los Pájaros (Seville, Spain; 6°14’W, 36°57’N), a private natural reserve with a small freshwater pond of about five hectares resulting from the restoration of an abandoned gravel pit and surrounded by ricefields. Cañada de los Pájaros concentrates a large diversity of free-living native birds and captive exotic and native birds and some mammals, including domestic animals and humans. As a part of an extensive study on the transmission of vector-borne diseases, from September to November 2012, 319 blood-fed mosquitoes were captured by direct aspiration while resting in the main building at Cañada de los Pájaros. Furthermore, 28 blood-fed mosquitoes were captured resting outside the building in the same locality and in other surrounding localities (i.e., Doñana National Park) using an aspirator, CDC-type downdraft miniature suction traps (model 1212; J. W. Hock, Gainesville, FL, USA) and BG traps (Biogents, Regensburg, Germany) supplemented with CO_2_. Subsequently, mosquitoes were enumerated on a chill table under a stereomicroscope to species levels using available morphological keys [21,22]. *Culex* mosquitoes belonging to the *univittatus* complex were identified as *Culex perexiguus* based on the criteria described by Harbach [23]. The digestion status of mosquito blood meals was scored visually according to the Sella score from zero (unfed mosquitoes) to seven (female without visible blood and eggs fully developed in their abdomen), following Detinova [24], see Figure 1. Mosquitoes were stored at −80°C until molecular analyses of blood meal origin were carry out.

### Blood meal identification

Mosquitoes were randomly assigned to one of two different DNA extraction protocols. The abdomens of 142 blood-fed mosquitoes were treated following the HotSHOT procedure: each abdomen was cut off using sterile tips and subsequently introduced into 75 μl of lysis solution (25 mM NaOH, 0.2 mM EDTA), crushed and incubated at 95°C for 30 minutes. After incubation, the solution was cooled on ice for five minutes and then 75 μl of neutralization solution (40 mM Tris–HCl) was added. At least two negative DNA extraction controls (i e, absence of blood) were included per plate. Abdomens were simultaneously processed using 96-thermowell plates and DNA extracts were stored at −20°C until PCR amplification. In addition, DNA from the abdomen of 205 blood-fed mosquitoes was isolated using the DNeasy Blood and Tissue® kit (QIAGEN, Hilden, Germany) following company specifications.

Vertebrate hosts were identified using a nested-PCR approach [25], which is effective to identify the feeding source of haematophagous insects. A fragment of 758 base pairs (bp) of the mitochondrial cytochrome oxidase 1 (COI) gen was amplified with the primary pair of primers M13BCV-FW and BCV-RV1 and the nested primer pair M13 and BCV-RV2. Positive amplifications were sequenced in one direction according to BigDye 1.1 technology (Applied Biosystems, Carlsbad, CA, USA). Labelled DNA fragments of PCR-positive products were resolved through an ABI 3130xl automated sequencer (Applied Biosystems, Carlsbad, CA, USA). Sequences were edited using the software Sequencher™ v4.9 (Gene Codes Corp, © 1991–2009, Ann Arbor, MI, USA) and assigned to particular vertebrate species when agreement was ≥98% to sequences of known species in GenBank DNA sequence database (National Center for Biotechnology Information Blast) or the Barcode of Life Data Systems (BOLD).

### Statistical analysis

A generalized linear mixed model (GLMM) with binomial distributed error and logit link function was used to test for the effect of the blood meal digestion status and the DNA extraction protocol on the success of host identification of blood-fed females. Identification success (0 or 1) was included as the dependent variable and DNA extraction protocol, Sella score and the interaction between both factors were included as independent variables. Mosquito species was included as a random factor and the model was fitted using Laplace approximation [26]. The analyses were repeated using only data from *An*. *atroparvus* (the species most extensively sampled) but given that the results were qualitatively the same, only the model including data from all species is shown. Analyses were done using SAS 9.2 (SAS Institute Inc 2010).

## Results

Overall, 347 blood-fed mosquitoes were included in this study. The most abundant species sampled was *An*. *atroparvus* followed by *Cx*. *perexiguus*, *Culex pipiens*, *Culex theileri*, *Culex modestus*, *Ochlerotatus caspius* and *Culiseta* sp. (Table 1). The blood meal source was successfully identified for 234 females (Table 1). The success of host identification decreased as the digestion status of the blood meal increased (F_5,330_=11.08, p<0.0001, Table 1). A higher success was obtained using the QIAGEN kit (F_1,330_=25.24, p<0.0001, 153/205, 74.6%) than the HotSHOT procedure (81/142, 57.0%). No interaction occurred between extraction method and digestion status (F_4,330_=1.43, p=0.23), although the differences in success with extraction method were not significant for females with very fresh blood meals (Sella score 2, F_1,330_=1.13, p=0.29).

**Table 1:** **Sella score of blood meal digestion**, **DNA extraction protocol used**, **and blood meal origin identification success for the 347 female mosquitoes analysed**

	**HotSHOT**					**QIAGEN kit**						
**Mosquito species**	**2**	**3**	**4**	**5**	**6**	**2**	**3**	**4**	**5**	**6**	**7**	**Total**
*Anopheles atroparvus*	19	15	17	10	16	27	20	20	16	8	3	171
*Culex modestus*	1	1	0	0	0	0	0	1	0	2	0	5
*Culex perexiguus*	40	3	2	1	1	8	8	7	5	7	2	84
*Culex pipiens*	3	3	1	2	1	9	6	4	4	7	3	43
*Culex theileri*	0	0	0	1	1	7	8	10	5	7	0	39
*Ochlerotatus caspius*	2	2	0	0	0	0	0	0	0	0	0	4
*Culiseta sp*.	0	0	0	0	0	0	0	0	0	1	0	1
Total	65	24	20	14	19	51	42	42	30	32	8	347
Identified blood meals	52	15	10	3	1	46	41	33	18	13	2	234
Success (%)	80.0	62.5	50.0	21.4	5.3	90.2	97.6	78.6	60.0	40.6	25.0	

Twenty-four host species were identified including four mammals, 14 birds and a single reptile (Table 2). DNA from an additional unidentified bird species was isolated. This unknown species could not be identified by direct comparison with those sequences deposited in Genbank. Rabbit sequences were confirmed by comparison with sequences isolated from fresh muscle tissue. In addition, three samples from *Anas* sp. and one sample from *Grus* sp. were identified to the genus level. Dog was the most common feeding source of mosquitoes compromising 71.4% of the identified blood meals. A single human derived blood meal from *An*. *atroparvus* was isolated. Evidence of mixed blood meals was not observed.

**Table 2:** Vertebrate host species identified for each mosquito species

**Mosquito species**	**Mammals**	**Birds**	**Reptiles**
*Anopheles atroparvus*	*Canis lupus familiaris* (99)	*Gallus gallus* (2)	
	*Equus caballus* (4)		
	*Equus asinus* (2)		
	*Ovis aries* (2)		
	*Oryctolagus cuniculus* (2)		
	*Rattus norvegicus* (2)		
	*Bos taurus* (1)		
	*Homo sapiens* (1)		
*Culex modestus*		*Anas sp*. (2)	
*Culex perexiguus*	*Canis lupus familiaris* (33)	*Gallus gallus* (9)	*Mauremys leprosa* (2)
	*Equus asinus* (1)	*Columba livia* (5)	
		*Passer domesticus* (1)	
		*Pterocles alchata* (1)	
		*Cygnus buccinator* (1)	
		*Corvus monedula* (1)	
		*Pauxi pauxi* (1)	
		*Anas sp*. (1)	
*Culex pipiens*	*Canis lupus familiaris* (7)	*Gallus gallus* (8)	
		*Passer domesticus* (4)	
		*Carduelis chloris* (2)	
		*Branta sandvicensis* (1)	
		*Cygnus atratus* (1)	
		*Grus sp*. (1)	
		Unidentified bird (1)	
*Culex theileri*	*Canis lupus familiaris* (28)	*Passer domesticus* (1)	
	*Cervus elaphus* (1)	*Haematopus ostralegus* (1)	
		*Meleagris gallopavo* (1)	
		*Gallus gallus* (1)	
*Culiseta sp*.		*Passer domesticus* (1)	
*Ochlerotatus caspius*	*Equus caballus* (2)		

Number of mosquitoes is recorded between brackets.

## Discussion

The efficiency of the analyses of host blood meal source differs widely between studies (i.e., 17.5%-92%, see [19,27]). In this study, the importance of two sources of variation was quantified. Digestion status of blood meals, visually estimated according to the Sella score, strongly affects the success of host identification using DNA sequencing with efficiencies ranging between 84.5% and 25.0% depending of digestion status. Similar results were obtained by [6] and [28] based on mosquitoes kept at the laboratory, with a significant decrease in the identification success 30 to 36 hours after feeding [6]. Although the time interval between insect feeding and collection was unknown in this study, for a recently fed individual (Sella stage 2) it may take about one day to reach the Sella stages 3 and 4, and 1 or 2 additional days to reach the Sella stages 5 and 6, respectively [29]. Results from the present study support those from previous studies where authors reported a reduction of the proportion of reactions yielding sequences as the Sella score on field-caught mosquitoes increased [30,31]. In this study, a significant drop in success of host identification was found for mosquitoes containing a blood meal in an advanced stage of digestion (Sella stages >5), a similar pattern found in mosquitoes from South Carolina [32]. Obviously, including mosquitoes with blood meals in the highest stages of digestion (scored as 5 and 7 according to the Sella´s method), the overall success of host identification may be reduced, and this may partially explain discrepancies between studies in the rate of host identification success. In addition, in this study, using QIAGEN kit for DNA extraction, the success of host identification significantly increased by 10.2-35.3% depending of the blood meal digestion status. The increase in performance was especially important for the mosquitoes with more digested blood meal (scored from 5 to 7 according to the Sella´s method). Using the QIAGEN kit 47% of blood meal sources was identified while only 12% of those extracted using the HotSHOT procedure was identified. This is also a higher percentage of success than those reported by Tuten et al. [32] where authors, using the DNAzol BD Direct Extraction Kit (Molecular Research Center, Cincinnati, OH, USA), identified 27% (6/22) blood meals from mosquitoes with 5 to 6 Sella´s scores. Consequently, large improvements in blood source determination may be obtained by using more efficient DNA extraction methods. This increase in efficiency is not obtained free as the economic cost of extraction per sample is much higher when using commercial kits, but the extra cost may be worth investing when the number of blood-fed females to analyse is limiting, as used to be the case in most vector ecology studies.

At least 25 vertebrate host species of mosquitoes potentially involved in the transmission of pathogens by mosquitoes have been identified. *Anopheles atroparvus* showed a clear preference to feed on mammals of different sizes, from rats to horses, than on avian species in spite of the presence of a high diversity and abundance of birds in the studied area, supporting results from previous studies [33,34]. Curiously, as recently reported, there is no information on the feeding preference of this species to bite indoors or outdoors [34]. Results obtained in this study clearly indicate that this species feed on surrounding animals located outdoors but use human-made shelters for resting after feeding, adding valuable information to current knowledge on the biology of this species [34]. On the other hand, *Cx*. *perexiguus*, the second more extensively sampled species in this study, fed on different bird species in addition to mammals and turtles. Its role as bird feeders, as is the case of other *Culex* species in this study, supports their importance in the transmission of wildlife diseases in Europe, i.e., West Nile and Usutu virus [9,18-20].

## Competing interests

The authors declare that they have no competing interests.

## Authors’ contributions

JMP SR RS JF conceived and designed the experiments, and contributed reagents/materials/analysis tools. All authors have read and approved the final manuscript.
